# Construction of an *in vitro* bypassed pyruvate decarboxylation pathway using thermostable enzyme modules and its application to *N*-acetylglutamate production

**DOI:** 10.1186/1475-2859-12-91

**Published:** 2013-10-07

**Authors:** Borimas Krutsakorn, Takashi Imagawa, Kohsuke Honda, Kenji Okano, Hisao Ohtake

**Affiliations:** 1 Department of Biotechnology, Graduate School of Engineering, Osaka University, 2-1 Yamadaoka, Suita, Osaka 565-0871, Japan; 2 PRESTO, Japan Science and Technology Agency (JST), 4-1-8 Honcho, Kawaguchi, Saitama 332-0012, Japan

## Abstract

**Background:**

Metabolic engineering has emerged as a practical alternative to conventional chemical conversion particularly in biocommodity production processes. However, this approach is often hampered by as yet unidentified inherent mechanisms of natural metabolism. One of the possible solutions for the elimination of the negative effects of natural regulatory mechanisms on artificially engineered metabolic pathway is to construct an *in vitro* pathway using a limited number of enzymes. Employment of thermostable enzymes as biocatalytic modules for pathway construction enables the one-step preparation of catalytic units with excellent selectivity and operational stability. Acetyl-CoA is a central precursor involved in the biosynthesis of various metabolites. In this study, an *in vitro* pathway to convert pyruvate to acetyl-CoA was constructed and applied to *N*-acetylglutamate production.

**Results:**

A bypassed pyruvate decarboxylation pathway, through which pyruvate can be converted to acetyl-CoA, was constructed by using a coupled enzyme system consisting of pyruvate decarboxylase from *Acetobacter pasteurianus* and the CoA-acylating aldehyde dehydrogenase from *Thermus thermophilus*. To demonstrate the applicability of the bypassed pathway for chemical production, a cofactor-balanced and CoA-recycling synthetic pathway for *N*-acetylglutamate production was designed by coupling the bypassed pathway with the glutamate dehydrogenase from *T. thermophilus* and *N*-acetylglutamate synthase from *Thermotoga maritima*. *N*-Acetylglutamate could be produced from an equimolar mixture of pyruvate and α-ketoglutarate with a molar yield of 55% through the synthetic pathway consisting of a mixture of four recombinant *E. coli* strains having either one of the thermostable enzymes. The overall recycling number of CoA was calculated to be 27.

**Conclusions:**

Assembly of thermostable enzymes enables the flexible design and construction of an *in vitro* metabolic pathway specialized for chemical manufacture. We herein report the *in vitro* construction of a bypassed pathway capable of an almost stoichiometric conversion of pyruvate to acetyl-CoA. This pathway is potentially applicable not only to *N*-acetylglutamate production but also to the production of a wide range of acetyl-CoA-derived metabolites.

## Background

The integration of diverse biocatalytic modules to expand the versatility of fermentation-based industries has been widely employed for the production of biofuel, pharmaceuticals, and other useful chemicals [1]. However, these “metabolic engineering” approaches often suffer from flux imbalances because the naturally occurring translational and transcriptional regulation mechanisms fail to appropriately function in artificially engineered cells [2,3]. One possible approach to overcome this limitation is to construct an *in vitro* metabolic pathway in which only a limited number of enzymes involved in the pathway-of-interest are used as catalytic modules. *In vitro* systems offer a number of potential advantages over the conventional fermentation-based production processes such as predictable production yield, operational flexibility, and scalability of the reaction [4,5]. However, the enzyme isolation generally requires laborious, costly, and time-consuming procedures, and thus little attention has been paid to the practical application of *in vitro* metabolic engineering approaches. Recently, we reported a simple approach to construct an *in vitro* artificial metabolic pathway using thermostable biocatalytic modules [6,7]. The basic procedure consists of the following four steps: 1) selection of suitable thermostable enzymes; 2) expression of the enzymes in mesophilic hosts (e.g. *Escherichia coli*); 3) preheating the cell suspension at high temperature; and 4) assembly of the catalytic modules at an experimentally optimized ratio to achieve stoichiometric production. The denaturation of indigenous enzymes at high temperatures results in a one-step preparation of highly selective and thermostable biocatalytic modules. The membrane structure of *E. coli* cells is partially or entirely disrupted at high temperatures, and consequently, better accessibility between the enzymes and substrates can be achieved [8].

Acetyl-CoA is a key precursor for the biosynthesis of a wide variety of industrially useful metabolites. In the mevalonate pathway, acetyl-CoA is carboxylated and conjugated to form various isoprenoid and terpenoid compounds that can be used as flavors, fragrances, and anticancer drugs [9]. Condensation of two acetyl-CoA units leads to the formation of acetoacetyl-CoA, which serves as a C4 backbone for polyhydroxyalkanoates and butanol biosynthesis [10-12]. Acetyl-CoA production tends to be a rate-limiting step of artificially engineered pathways to produce target compounds because acetyl-CoA serves a central intermediate of a wide range of naturally occurring metabolic pathways and thus is subjected to depletion when it is routed into co-existing pathways [2].

In natural metabolism, acetyl-CoA is mainly generated from pyruvate via three routes. Most aerobic microorganisms use the large protein complex of pyruvate dehydrogenase (PDH) to produce acetyl-CoA [13]. Alternatively, pyruvate-ferredoxin oxidoreductase (PFOR) plays an important role in the oxidative decarboxylation of pyruvate in some anaerobic bacteria and hyperthermophilic achaea [14-16]. Pyruvate formate-lyase (PFL) is another key enzyme involved in the carbohydrate metabolism of anaerobic microorganisms [17,18]. However, none are readily available in *in vitro* production systems. PDH is one of the largest protein complexes and consists of multiple copies of three or four subunits [19]. For example, the PDH of *E. coli* consists of a central cubic core composed of 24 molecules of dihydrolipoamide acetyltransferase (E2), onto which up to 24 copies of pyruvate dehydrogenase (E1) and 12 copies of dihydrolipoamide dehydrogenase (E3) are assembled [19]. To our knowledge, there have been no reports on the functional expression of the complete form of PDH in heterologous hosts probably owing to their highly complex nature. PFORs require specific redox partner proteins, namely ferredoxin and ferredoxin reductase, to exert their catalytic abilities. In *in vitro* systems, the enzymes and the partner proteins freely diffuse in the reaction mixture, resulting in less frequent interactions between them. In addition, the poor oxygen tolerance of PFOR results in operational limitations for its *in vitro* use. Similarly, PFL is highly sensitive to oxygen stress [20].

To overcome these limitations, in this study, we constructed an *in vitro* bypassed pathway capable of converting pyruvate to acetyl-CoA using an enzyme couple of thermostable pyruvate decarboxylase and CoA-acylating aldehyde dehydrogenase. Owing to the absence of a co-existing pathway, an almost stoichiometric production of acetyl-CoA could be achieved through the *in vitro* pathway. Furthermore, the performance of this pathway was assessed by integration to an artificial pathway for the production of *N*-acetylglutamate (NAG), which can be used as an ingredient in the cosmetic industry as an anti-odor compound and skin moisturizer [21,22].

## Results

### Construction of the bypassed pyruvate decarboxylation pathway

**Figure 1:**
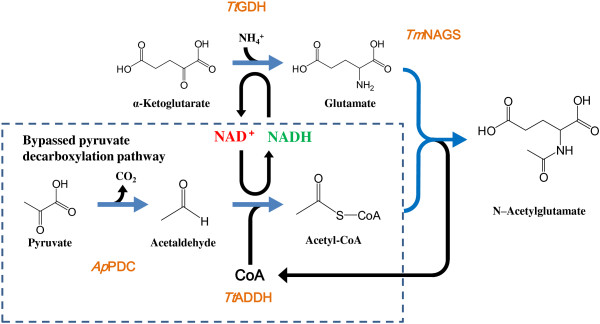
**Schematic illustration of the designed pathway for NAG production.** Abbreviations used: *Ap*PDC, pyruvate decarboxylase from *Acetobacter pasteurianus*; *Tt*ADDH, CoA-acylating aldehyde dehydrogenase from *Thermus thermophilus*; *Tt*GDH, glutamate dehydrogenase from *Thermus thermophilus*; *Tm*NAGS, *N*-acetylglutamate synthase from *Thermotoga maritima*.

**Figure 2:**
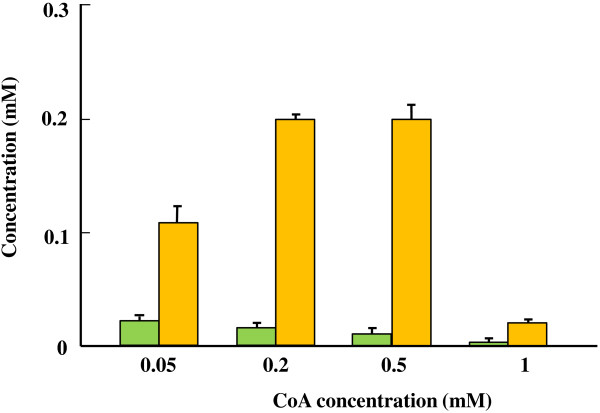
**Effect of CoA concentrations on the reaction specificity of *Tt*ADDH.** The reaction was performed at 50°C in the mixture composed of 50 mM HEPES-NaOH (pH 7.0), 1 mM NAD^+^, 1 mM acetaldehyde, approximately 0.16 U ml^-1^*Tt*ADDH, and the indicated concentrations of CoA. After the preincubation for 1 min at 50°C, the reaction was initiated by the addition of acetaldehyde. After the incubation at 50°C for 1 min, the reaction was quenched by adding an equal volume of ice-cold 0.5 N HCl in methanol. The mixture was analyzed by HPLC to quantify acetate (green bar) and acetyl-CoA (yellow bar).

A bypassed pyruvate decarboxylation pathway was designed by using an enzyme couple of pyruvate decarboxylase and CoA-acylating aldehyde dehydrogenase (Figure 1). Among microbial pyruvate decarboxylases, those from mesophilic bacteria, including *Zymomonas mobilis*[23] and *Acetobacter pasteurianus*[24], are biochemically well characterized. Although BLAST searches of the genomic sequences of (hyper)thermophiles gave no hits when the amino acid sequences of these enzymes were used as queries, the pyruvate decarboxylase from *A. pasteurianus* (*Ap*PDC) has been reported to have a relatively high thermal stability with a half-life of 2 h at 60°C [24,25]. The CoA-acylating aldehyde dehydrogenase was obtained from those involved in the gene-expression library of *Thermus thermophilus* HB8 [26] and designated as *Tt*ADDH. We found that *Tt*ADDH could catalyze the oxidation of acetaldehyde in both CoA-dependent (acetyl-CoA-forming) and CoA-independent (acetate-forming) manners (Figure 2). Increased CoA concentration (up to 0.5 mM) led to the shift in the reaction specificity of the enzyme to the acetyl-CoA-forming direction (Figure 2). On the other hand, CoA concentration higher than 0.5 mM caused a marked inhibition of the enzyme activity. The CoA concentration of 0.2 mM was used in further studies since no significant difference was observed in the reaction specificity and the activity of *Tt*ADDH at CoA concentrations of 0.2 and 0.5 mM.

**Figure 3:**
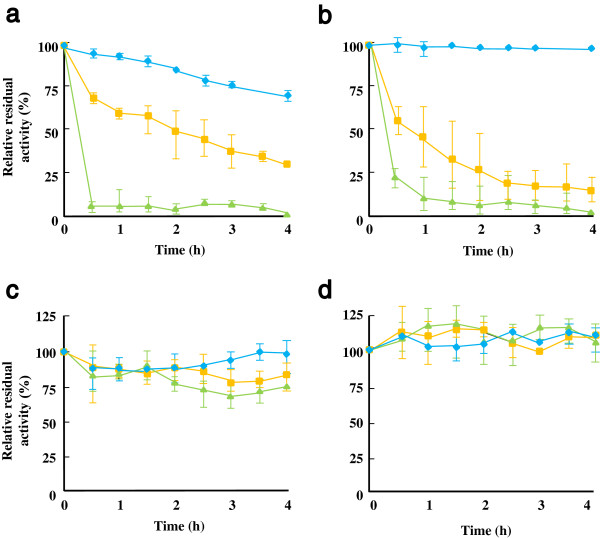
**Thermal stability of *Ap*PDC (a), *Tt*ADDH (b), *Tt*GDH (c), and *Tm*NAGS (d).** Crude lysates of the recombinant *E. coli* were preheated at 70°C for 30 min; an incubation temperature of 60°C was employed for preheating the lysate having *Ap*PDC. After the removal of denatured proteins by centrifugation, the enzyme solutions were incubated at 50°C (blue diamond), 60°C (yellow square), and 70°C (green triangle) for the indicated time periods. Residual enzyme activity was determined using the standard assay conditions.

*Ap*PDC could retain more than 60% of its activity after incubation at 50°C for 4 h, whereas no apparent decrease was observed in the activity of *Tt*ADDH at 50°C (Figure 3a and b). Conversely, the activities of both enzymes steeply declined at an incubation temperature of 60°C or higher. Thus, a reaction temperature of 50°C was used for further studies.

**Figure 4:**
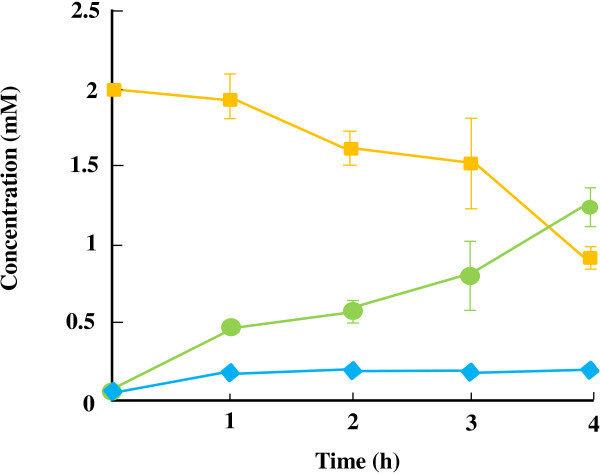
**Acetyl-CoA production through the bypassed pyruvate decarboxylation pathway.** Heat-treated cells of recombinant *E. coli* having *Ap*PDC and *Tt*ADDH (approximately 8 and 4 mg ml^-1^, respectively) were incubated at 50°C with 2 mM each of pyruvate, NAD^+^, and CoA. Acetyl-CoA (green circle), CoA (yellow square) and acetate (blue diamond) concentrations were quantified by HPLC.

When the heat-treated cells having *Ap*PDC and *Tt*ADDH were incubated with an equimolar mixture of pyruvate, NAD^+^, and CoA (2 mM each) at 50°C, acetyl-CoA could be produced with a molar yield of 65% (Figure 4). A relatively slow production rate observed in the initial 2 h is probably attributed to the inhibitory effect of the high initial concentration of CoA on *Tt*ADDH.

### Design of the NAG production pathway

The bypassed pyruvate decarboxylation pathway was integrated into a newly designed synthetic pathway for NAG production to evaluate its applicability to chemical production (Figure 1). In the designed pathway, NAG is produced through transacetylation from acetyl-CoA to glutamate catalyzed by *N*-acetylglutamate synthase (NAGS). CoA molecules released by the acetylation of glutamate can be recycled for the synthesis of acetyl-CoA in the bypassed pyruvate decarboxylation pathway. The thermostable NAGS, which is involved in the arginine biosynthesis pathway of *Thermotoga maritima*, is well characterized [27], therefore, the NAGS from *T. maritima* was selected as the module to construct the pathway and designated *Tm*NAGS.

Unlike natural metabolic pathways, which are equipped with a complete enzyme apparatus for the *de novo* synthesis of cofactors, indigenous regeneration of redox cofactors is necessary to facilitate the economic feasibility of *in vitro* production processes [4,6]. To achieve balanced NAD^+^/NADH consumption and regeneration, the glutamate dehydrogenase of *T. thermophilus* (*Tt*GDH) was integrated into the NAG production pathway. The enzyme can use α-ketoglutarate, an inexpensive material, as a cosubstrate for the re-oxidation of NADH and to produce glutamate, which can be directly used as the substrate for *Tm*NAGS. Thermal stability analysis of *Tm*NAGS and *Tt*GDH demonstrated that they can be used at 50°C for at least 4 h without a significant loss of their activities (Figure 3c and d).

### NAG production

Activities of *Ap*PDC, *Tt*ADDH, *Tt*GDH, and *Tm*NAGS were assessed under the standard assay conditions at a substrate concentration of 0.2 mM. One unit (U) of either enzyme was defined as the amount capable of product formation at a rate of 1.0 μmol min^-1^. Enzyme activities were assessed at various pHs using the crude extracts of recombinant *E. coli* cells (Table 1). The pH profiles of the enzymes revealed that the total protein concentration in the mixture of the crude extracts containing 0.04 U ml^-1^ of each enzyme can be minimized at pH 7.0.

**Table 1:** pH profile of enzyme activity

**Buffer**	**pH**	**Specific activity (U mg**^**-1 **^**total protein)**^**a**^				**Total protein concentration (mg ml**^**-1**^**)**^**b**^
		***Ap*PDC**	***Tt*ADDH**	***Tt*GDH**	***Tm*NAGS**	
MES	6.0	0.046	0.067	0.164	0.154	1.97
MES	6.5	0.066	0.146	0.255	0.169	1.27
HEPES	7.0	0.078	0.137	0.291	0.208	1.13
HEPES	7.5	0.058	0.197	0.184	0.215	1.30
Bicine	8.0	0.046	0.246	0.074	0.220	1.75
Bicine	8.5	0.025	0.450	0.026	0.227	3.37

^a^Specific activities are expressed as those from the crude extract of recombinant *E. coli* before the heat treatment.

^b^Total protein concentrations in the mixture of crude extracts containing 0.04 U of each of the enzyme are calculated.

The flux through the bypassed pyruvate decarboxylation pathway could be determined by incubating an enzyme mixture of *Ap*PDC and *Tt*ADDH with 0.2 mM each of pyruvate, CoA, and NAD^+^, and monitoring the concomitant production rate of NADH at 340 nm. When equal units (0.04 U ml^-1^) of the enzymes were used, the initial NADH production rate was lower than the expected value of 0.04 μmol min^-1^ ml^-1^. In the constructed pathway, the actual concentration of acetaldehyde, which is generated by *Ap*PDC and then used as the substrate for *Tt*ADDH, was kept at a lower level than those used in the standard assay, and thus *Tt*ADDH failed to catalyze the reaction at the expected rate. We increased the *Tt*ADDH concentration in the reaction mixture in a stepwise manner, and found that the expected NADH production rate of 0.04 μmol min^-1^ ml^-1^ could be achieved by using 0.09 U ml^-1^ of *Tt*ADDH.

**Figure 5:**
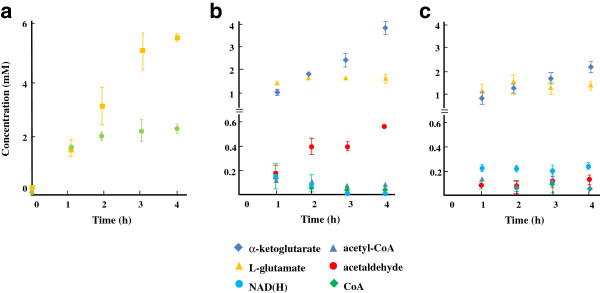
**Time course analysis of NAG production. (a)** Time profiles of NAG production. Reaction was performed with (yellow square) and without (green circle) NADH feeding at a rate of 3.3 μmol l^-1^ min^-1^. **(b, and c)** Time profiles of the cofactors and intermediates during NAG production in the absence **(b)** and presence **(c)** of NADH feeding. The concentration of pyruvate was under the detectable level.

The direct production of NAG from pyruvate and α-ketoglutarate was performed by using a mixture of heat-treated recombinant cells having the experimentally determined amounts of enzymes to achieve a production rate of 0.04 μmol ml^-1^ min^-1^ (i.e. 0.04 U ml^-1^ each of *Ap*PDC, *Tt*GDH, and *Tm*NAGS, and 0.09 U ml^-1^ of *Tt*ADDH). The reaction was performed in a mixture containing 0.2 mM each of pyruvate, α-ketoglutarate, L-glutamate, and CoA. A mixture of pyruvate, α-ketoglutarate, and NH_4_Cl was continuously supplied into the reaction mixture at a rate of 0.04 μmol ml^-1^ min^-1^ to keep a constant flux. However, NAG production stopped after 2 h despite the sufficient thermal stabilities of the enzymes (Figure 5a). The time profile analysis of the pool sizes of the intermediates and cofactors revealed the concentrations of the redox cofactors, NAD^+^ and NADH, decreased time dependently while those of acetaldehyde and α-ketoglutarate remarkably increased (Figure 5b). These facts implied that the thermal decomposition of the redox cofactors led to the decrease in the catalytic performance of NAD(H)-dependent enzymes, *Tt*ADDH and *Tt*GDH. Moreover, owing to its highly volatile nature, acetaldehyde accumulation likely led to the loss in the NAG production yield through the constructed pathway. On the basis of these observations, a NAG production assay was conducted with a continuous feeding of NADH at a rate identical to that of the thermal decomposition (3.3 nmol ml^-1^ min^-1^). Consequently, 5.3 mM of NAG could be produced with a molar yield of 55% after a 4-h reaction. The total recycling number of CoA was calculated to be 27. The time profile of intermediates confirmed that the accumulation of α-ketoglutarate and acetaldehyde could be mitigated by the addition of NADH (Figure 5c).

## Discussion

Acetyl-CoA is an essential intermediate for the production of a variety of metabolites; therefore intensive studies have been conducted to increase the intracellular level of acetyl-CoA in artificially engineered microorganisms. Atsumi *et al.* reported that the increases in the intracellular pool size of acetyl-CoA, which can be achieved by deleting the gene sets of *ldh*A, *adh*E, and *frd*BC of *E. coli*, leads to a two-fold increase in 1-butanol production by the engineered cells [2]. Shiba *et al.* have engineered a pyruvate dehydrogenase bypass pathway in *Saccharomyces cerevisiae* to increase intracellular acetyl-CoA and to improve the production level of amorphadiene, a sesquiterpene precursor for the biosynthesis of the anti-malarial drug artemisinin [28]. Furthermore, Chen *et al*. have developed an engineered yeast platform for improved acetyl-CoA production and demonstrated its application for α-santalene production [12].

Previously, we reported a simple approach to construct an *in vitro* artificial metabolic pathway using thermostable biocatalytic modules [6,7]. This approach is, in principle, applicable to any thermostable enzyme as long as it can be functionally expressed in a mesophilic host, and thus is potentially applicable to the biocatalytic manufacture of a wide range of useful compounds. By using this approach, we have constructed a non-ATP-forming chimeric glycolytic pathway and applied it to the direct conversion of glucose to lactate [6] and malate [7]. The construction of an *in vitro* pyruvate decarboxylation pathway to form acetyl-CoA was one of the key challenges remaining to expand the applicability of this system.

**Figure 6:**
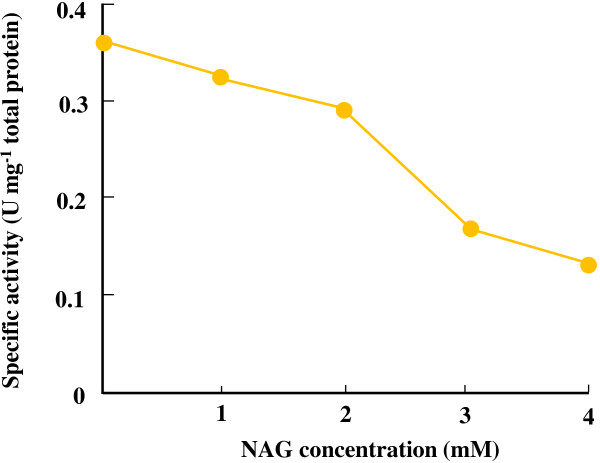
**Effect of NAG concentration on *Tt*GDH.** The enzyme activity was determined under the standard assay conditions at the indicated concentrations of NAG.

In this study, we constructed an *in vitro* bypassed pathway for pyruvate decarboxylation by employing an enzyme couple of *Ap*PDC and *Tt*ADDH. The bypassed pathway was integrated into the newly designed synthetic pathway for NAG production, in which consumption and regeneration rates of both NAD(H) and CoA were essentially balanced. The highly traceable nature of the *in vitro* synthetic pathway enabled us to quantify the intermediate pool sizes without using special equipment and to identify the rate-limiting of the synthetic pathway. The time-course analysis of the intermediates in the NAG-producing pathway indicated that the decrease in the catalytic ability of *Tt*GDH and *Tt*ADDH caused by the thermal decomposition of NAD(H) was the bottleneck. In fact, by continuously supplying NADH to the reaction mixture, 5.4 mM NAG could be produced with a molar yield of 55% and a CoA-recycling number of 27 could be achieved. The operational stability of the *in vitro* production system would be improved by the screening and employment of a thermostable glutamate dehydrogenase with lower *K*_m_ for NAD(H). However, the intermediate analysis indicated that accumulation of α-ketoglutarate was still not insignificant even when NADH was supplied (Figure 5c). This can be explained by the inhibitory effect of NAG on *Tt*GDH (Figure 6). Although *in vitro* synthetic pathways are independent of transcriptional and translational regulatory mechanisms of living cells, allosteric regulation cannot be necessarily eliminated. Protein engineering approaches or the substitution with another enzyme module that is less sensitive to allosteric effects would be a straightforward way to eliminate the effect of allosteric inhibition. In fact, we previously demonstrated that the inhibitory effect of NAD^+^ on lactate production through the chimeric glycolytic pathway can be eliminated by changing the NAD^+^-sensitive lactate dehydrogenase to malate/lactate dehydrogenase [6]. Use of a NAG-insensitive thermophilic glutamate dehydrogenase would be needed to achieve a higher titer of NAG production through the synthetic pathway.

Another possible approach to mitigate the effect of product inhibition is the employment of a continuous product recovery system. Recently, we reported that the pretreatment of recombinant *E. coli* cells having a thermophilic fumarase with a low concentration glutaraldehyde (~0.15 vol%) can consolidate the membrane structure of the cells and prevent the heat-induced leakage of the enzyme [8]. The glutaraldehyde-treated cells can be used at 70°C for more than 10 h in a continuous reactor equipped with a cell-separation membrane filter. In the conversional fermentation-based processes, the culture broth contains a number of impurities including residual sugars, nutrients, and other organic materials, apart from cell mass. By contrast, in *in vitro* production systems, the reaction can be performed in a simple buffer solution, and thus the product separation step can be considerably simplified. Development and integration of an on-line product separation system would be a key challenge to eliminate product inhibition and to achieve the long-term operation of the *in vitro* production system.

## Conclusions

We designed and constructed an *in vitro* pyruvate decarboxylation pathway capable of converting pyruvate to acetyl-CoA using an enzyme couple of *Ap*PDC and *Tt*ADDH. To demonstrate the applicability of the bypassed pathway to chemical production, the pathway was coupled with *Tt*GDH and *Tm*NAGS to construct a cofactor-balanced and CoA-recycling pathway for NAG production. The highly traceable nature of the *in vitro* pathway enabled us to readily identify the rate-limiting enzyme. Consequently, 5.4 mM of NAG could be produced through the pathway with a molar yield of 55% and a CoA-recycling number of 27. The bypassed pathway constructed in this study would be widely applicable to the production of other acetyl-CoA-derived chemicals including *n*-butanol, polyhydroxyalkanoates, and isoprenoids.

## Materials and methods

### Bacterial strain and plasmid

The expression plasmids encoding *Tt*ADDH (Genebank accession number, YP_145486.1) and *Tt*GDH (YP_144842.1) were obtained from the RIKEN *T. thermophilus* HB8 expression plasmid set [26]. The gene coding for *Ap*PDC (YP_005499956.1) was amplified by PCR from the chromosomal DNA of *A. pasteurianus* using the following primers: 5′-AA*CATATG*ACCTATACTGTTGGCAT-3′ (the *Nde*I restriction site is shown in italics) and 5′-TT*CTCGAG*TCAGGCCAGAGTCGTCTTGC-3′ (the *Xho*I restriction site is shown in italics). The amplified DNA was digested with *Nde*I and *Xho*I and inserted into the corresponding restriction sites of pET21a (Novagen, Madison, WI, USA). Similarly, the gene encoding *Tm*NAGS (NP_229580.1) was amplified from the chromosomal DNA of *T. maritima* using a primer pair of 5′-AA*CATATG*TTCACTCCCAGGGGTTT-3′ (the *Nde*I restriction site is shown italics) and 5′-TT*GAATTC*TCATGTTCTGTACCTCCCGT-3′ (the *EcoR*I restriction site is shown italics), and then introduced into pET21a. The expression vectors were separately introduced in *E. coli* Rosetta2 (DE3) (Novagen). Recombinant cells were cultivated aerobically at 37°C in Luria-Bertani (LB) media supplemented with 50 μg ml^-1^ ampicillin and 30 μg ml^-1^ chloramphenicol. Isopropyl-β-D-1-thiogalactopyranoside was added to the culture at a final concentration of 0.2 mM in the late-log phase. The cells were harvested by centrifugation and washed once with 50 mM 4-(2-hydroxyethyl)-1-piperazineethanesulfonic acid (HEPES)-NaOH buffer (pH 7.0).

### Enzyme preparation

Recombinant cells were suspended in an appropriate volume of 50 mM HEPES-NaOH (pH 7.0) and disrupted using an UD-201 ultrasonicator (Kubota, Osaka, Japan) at 80 W for 2 min. Cell debris were removed by centrifugation at 12,000 × *g* at 4°C for 10 min. Protein concentration in the crude lysates was measured using a Bio-Rad assay system (Bio-Rad, Hercules, CA, USA) with bovine serum albumin as the standard. The resulting crude lysates were incubated at 70°C for 30 min to inactivate the indigenous enzymes. An incubation temperature of 60°C was employed for the heat treatment of the lysate with *Ap*PDC. The heat-treated lysates were centrifuged again to remove the denatured protein and then the supernatants were used as enzyme solutions.

### Enzyme assay

All assays were performed at 50°C in 50 mM HEPES-NaOH (pH 7.0) supplemented with 100 mM KCl and 10 mM MgCl_2_ unless otherwise stated. Activities of *Ap*PDC, *Tt*ADDH, and *Tt*GDH were spectrophotometrically determined by monitoring the consumption or formation rate of NADH at 340 nm. *Tt*ADDH was assayed in the buffer containing 0.2 mM NAD^+^, 0.2 mM CoA, and an appropriate amount of the enzyme. After the preincubation at 50°C for 2 min, the reaction was started by the addition of acetaldehyde at a final concentration of 0.2 mM, and the reduction of NAD^+^ was monitored at 340 nm. The activity of *Tt*GDH was assessed in a similar manner by monitoring the oxidation of NADH upon the reduction of 0.2 mM α-ketoglutarate. *Ap*PDC activity was assessed by coupling with *Tt*ADDH. After the preincubation of the enzymes at 50°C in the buffer containing 0.2 mM NAD^+^, 0.2 mM CoA, and an excess amount of *Tt*ADDH, the reaction was initiated by the addition of 0.2 mM pyruvate and monitored at 340 nm.

*Tm*NAGS activity was assessed by determining the NAG production rate by high-performance liquid chromatography (HPLC). The appropriate amount of enzyme solution was incubated at 50°C in the buffer containing 0.2 mM each of L-glutamate and acetyl-CoA. The reactions were quenched by the addition of an equal volume of ice-cold methanol. HPLC analysis was performed as described below. One unit (U) of the enzyme was defined as the amount capable of product formation at a rate of 1 μmol min^-1^.

### NAG production

The basal reaction mixture was composed of 100 mM KCl, 10 mM MgCl_2_, 0.2 mM CoA, 0.2 mM NAD^+^, 0.2 mM NADH, and 50 mM HEPES-NaOH (pH 7.0). Recombinant cells were suspended in 50 mM HEPES-NaOH (pH 7.0) and heated at 70°C for 30 min, although an incubation temperature of 60°C was employed for the heat-treatment of *E. coli* having *Ap*PDC. The heat-treated suspensions of *E. coli* having *Ap*PDC, *Tt*ADDH, *Tt*GDH, and *Tm*NAGS were added into the reaction mixture at final concentrations of 16, 7, 50, and 10 mg (wet weight cells) ml^-1^, respectively. The mixture (8 ml) was placed in a 10-ml rubber-capped cylindrical vessel and stirred in a water bath kept at 50°C. The reaction was initiated by adding the indicated concentrations of substrates and the intermediates. The feeding of a substrate solution consisting of 100 mM each of α-ketoglutarate, pyruvate, and NH_4_Cl was started using a LC-20 AD solvent delivery unit (Shimadzu, Kyoto, Japan). The feeding rate was kept at 3.2 μl min^-1^ (= 0.04 μmol ml^-1^ min^-1^). Simultaneously, 0.2 mM pyruvate, 0.2 mM acetyl-CoA, 0.2 mM α-ketoglutarate, 0.2 mM L-glutamate, and 100 mM NH_4_Cl were added to the reaction mixture to maintain the constant pool sizes. The reaction was quenched at timed intervals by the addition of an equal volume of pre-cooled methanol and analyzed to determine the product and intermediates.

### Analytical methods

Acetate, α-ketoglutarate, pyruvate, and NAG were determined by HPLC on two ion exclusion columns connected in tandem as described previously [6]. CoA and acetyl-CoA were quantified by HPLC using a Cosmosil 5C18-ARII column (4.6 × 250 mm, Nacalai Tesque, Kyoto, Japan). The column was eluted with 0.1% (v/v) orthophosphate solution for the initial 2 min and then with a linear gradient of methanol (0–30 vol%) for 10 min. The column was maintained at 40°C and the flow rate of 1.0 ml min^-1^ was employed for the elution. The eluent was analyzed with a SPD-20AV UV-VIS detector (Shimadzu) at 254 nm. Aldehydes were fluorescence-labeled with 1,3-cyclohexanedione as described elsewhere [29] and determined using HPLC on the same column. The column was eluted at 40°C using 30% (v/v) acetonitrile at a flow rate of 0.5 ml min^-1^. The eluent was analyzed with an RF10A fluorometric detector (Shimadzu) at excitation and emission wavelengths of 366 and 440 nm, respectively. NAD(H) and glutamate were quantified using commercial kits as described in the instructions provided by the manufacturers (Biovision, Mountain View, CA, USA and Cosmo Bio, Tokyo, Japan, respectively).

## Competing interests

The authors declare that they have no competing interests.

## Authors’ contributions

BK performed the experiments and wrote the manuscript. TI constructed the bypassed pyruvate decarboxylation pathway. KH designed all the experiments and wrote the manuscript. KO contributed general advice, particularly on the thermophilic microorganisms, as well as edited the manuscript. HO supervised the work. All authors read and approved the final manuscript.
